# Maternal anemia and risk of adverse maternal health and birth outcomes in Bangladesh: A nationwide population-based survey

**DOI:** 10.1371/journal.pone.0277654

**Published:** 2022-12-16

**Authors:** Md. Awal Kabir, Md. Mostafizur Rahman, Md. Nuruzzaman Khan

**Affiliations:** 1 Department of Social Work, Pabna University of Science and Technology, Pabna, Bangladesh; 2 Department of Population Science and Human Resource Development, University of Rajshahi, Rajshahi, Bangladesh; 3 Department of Population Science, Jatiya Kabi Kazi Nazrul Islam University, Mymensingh, Bangladesh; Medical Research Council, SOUTH AFRICA

## Abstract

**Background:**

Maternal anemia is an ongoing public health challenge in low- and middle- income countries, including Bangladesh. The aim of this study was to explore the association of maternal anemia with a range of adverse maternal health and birth outcomes in Bangladesh.

**Methods:**

A total of 2,259 maternal women data was analyzed, extracted from the 2011 Bangladesh Demographic and Health Survey. Outcome variables considered were a range of maternal health and birth outcomes. Adverse maternal health outcomes were pregnancy complications, pregnancy termination, menstrual irregularities, cesarean delivery, diabetes, and hypertension. Adverse birth outcomes considered were low birth weight, stillbirths, early neonatal deaths, perinatal deaths, preterm birth, and prolonged labor. The main exposure variable was maternal anemia status. Mixed effect multilevel logistic/poisson regression model was used to determine the association between exposure and outcome variable adjusted for individual-, household-, and community-level factors.

**Results:**

The reported prevalence of anemia was 44%. A higher likelihoods pregnancy complication (AOR, 1.39, 95% CI, 1.09–2.41, p<0.05) and lower likelihoods of menstrual irregularities (AOR, 0.79, 95% CI, 0.58–0.94, *p<0*.*05*), diabetes (AOR, 0.78, 95% CI, 0.49–0.98, *p<0*.*05*) and hypertensive (AOR, 0.79, 95% CI, 0.60–0.96, *p<0*.*05*) were found among anemic maternal women as compared to the non-anemic maternal women. Adverse birth outcomes, including preterm birth (AOR, 2.03, 95% CI, 1.01–4.25, *p<0*.*05*), early neonatal mortality (AOR, 1.87, 95% CI, 1.06–5.10), and perinatal mortality (AOR, 1.54, 95% CI, 1.09–3.52, *p<0*.*05*), were also found higher among newborn of anemic maternal women as compared to the newborn of non-anemic maternal women.

**Conclusion:**

Anemia during pregnancy increases the occurrence of adverse maternal health and birth outcomes. Strategies to reduce anemia, such as iron supplementation, during pregnancy and among reproductive-aged women need to be prioritized in the policies and programs.

## Introduction

Maternal anemia (hemoglobin concentration (<110 g/dl)) during pregnancy is common worldwide with around 25% prevalence [1]. It is even found higher in Low- and Middle-Income Countries (LMICs, 42%), especially in Central and West Africa (56%) and in South Asia (52%) [2]. Such a higher prevalence of maternal anemia in LMICs has been found to be linked with several adverse maternal health and birth outcomes. For instance, there is an estimate that anemia during pregnancy is responsible for around 20% of total maternal deaths worldwide [3, 4]. It is also estimated that anemia causes 591,000 perinatal deaths worldwide [5], a majority of them are occurred in LMICs, particularly in South Asia and Africa [6]. It is also found that, maternal anemia increases the occurrence of low birth weight and preterm birth. These are major causes of neonatal and infant mortality in LMICs [6–9], though contradiction over this association is reported in few other studies [10, 11]. Anemia during pregnancy reduces maternal gestational age which lead to increase occurrence of low- birth weight and preterm births [12] as well as other others adverse outcomes [6, 13]. Because of these higher adverse maternal and birth outcomes among anemic maternal women, maternal anemia is considered an ongoing public threat in LMICs and a challenge to achieving the health-related Sustainable Development Goals (SDGs) by 2030, particularly goals related to maternal (target 3.1) and child health (target 3.2) [14].

Bangladesh, a LMIC, has been observed significant improvement in several reproductive, maternal and child health indicators over the years, particularly during the MDGs period of 2000 to 2015 [15]. Many other LMICs have been observed a similar improvement [16, 17]. However, the occurrences of adverse maternal and child health outcomes are still very high. Particularly, the current trend of improvement is significantly inadequate as compared to the requirements that we need to achieve the relevant SDGs’ targets by 2030. Many of these adverse outcomes, including particularly fetal death [18] and low birth weight [19], have been found to be linked with maternal anemia during pregnancy. Consequently, the Bangladesh’s government is now prioritizing policies and programs to reduce the prevalence of anemia in order to achieving the SDGs’ target by 2030 as well as achieving the global nutrition target by 2025 [20]. However, Bangladesh is still far beyond to achieving these targets and the country still facing a higher burden due to anemia during pregnancy.

Previous studies in Bangladesh, as well as LMICs, have explored several individual and household level factors associated maternal anemia. However, adverse effects of maternal anemia on health and birth outcomes have not been explored broadly yet, both in Bangladesh and in LMICs. Available studies have mainly been examined the effects of maternal anemia on low birth weight, preterm birth, and small gestational age [6, 7], though their findings were inconsistent. As such, systematic review and meta-analysis based on these published studies have also been reported inconsistent findings [8–11]. A mixed finding was also reported for maternal anemia and small for gestational age as well [6, 12, 13], a factor which is found to be linked with preterm birth and low birth weight. The reasons for such differences are the analysis of a small set of data, collected regionally or in the healthcare setting, imprecise analysis technique and consideration of an inadequate list of confounding factors. However, such contradiction in findings put the policy makers in dilemma over the degree of adverse effects of maternal anemia on adverse birth and health outcomes.

Previous studies in Bangladesh have mainly been examined the prevalence and risk factors of maternal anemia [21–23]. A few studies have also been focused on the impact of iron supplementation on reduction of anemia prevalence [24, 25] and reduction of adverse perinatal outcomes, including fetal deaths [18], and low birth weight [19]. However, none of previous studies examined all possible birth and health outcomes in relation to maternal anemia using population-based survey data. We conducted this study to fill these gaps. We explored the effects of maternal anemia on a range of adverse birth and maternal health outcomes by analyzing the nationally representative population-based survey data.

## Methods

### Study design and sampling

We analysed 2011 Bangladesh Demographic and Health Survey (BDHS) data [26]. It is a nationally representative household survey conducted in every three years as part of the Demographic and Health Survey Program of the USA. The households were selected by using two-stage stratified random sampling methods. Total of 600 clusters were selected at the first stage of sampling. The list of clusters generated by the Bangladesh Bureau of Statistics to conduct the 2001 National Population Census was used for this purpose. A fixed number of 30 households was selected at the second stage of sampling through probability proportional to the cluster size. Reproductive-aged married women who are the usual resident of the selected households or passed the most recent night at the selected households were primary respondents of the survey. This produced a list of 18,222 women to be included in the survey. Of them, 17,842 maternal women were included in the survey. Anthropometric data were recorded for 5,902 women. Details of this survey sampling procedure have been published elsewhere [26].

### Analytical sample

The primary inclusion criteria of this study were maternal women who gave at least one birth within five years preceding the survey. As such, women having no children (n = 573) and who have not had any birth within five years of the survey date (n = 2,845) were excluded. Respondents with missing data in any of the considered outcomes, covariates and/or exposure variables (n = 225), as presented in the two subsections below, were also excluded. The final analysis sample consisted of 2,259 maternal women.

### Exposure variable

Anemia status was considered as the exposure variable. The survey measured and recorded haemoglobin data by following the standard guideline available in the DHS methodological report [27]. We used this data and reclassified maternal women as anemic (if hb values <110 g/dl) and non-anemia (if hb values ≥110 g/dl) as per the World Health Organization (WHO) guideline [28].

### Outcomes variables

A range of maternal health and adverse birth outcomes was considered as outcome variables. Maternal health outcomes were pregnancy complications (symptoms, e.g., bleeding, severe nausea and vomiting, that adversely affect the health of pregnant women and warrant immediate medical attention), pregnancy termination (termination of the embryo or fetus before capable of independent life or survival outside the uterus), menstrual irregularities (menstrual irregularities occurs when its cycle vary from normal menstrual cycle ranged 21–34 days), cesarean delivery (method childbirth in which a surgical incision is made through a mother’s abdomen and uterus to deliver the baby), diabetes (FBG ≥7.0 mmol/L OR self-reported diabetes medication), and hypertension (SBP≥140 mmHg/ DBP≥90 mmHg OR taking antihypertensive medication), Low birth weight (babies born with weight <2500 gm), prolonged labor (duration of labor pain is more than 12 hours), preterm birth (birth before 37 weeks of gestation), stillbirths (stillbirths are fetal deaths in pregnancies lasting seven or more months.), early neonatal mortality (deaths at age 0–7 days), perinatal mortality (sum of the number of stillbirths and early neonatal deaths is perinatal mortality) were included as birth outcomes.

### Covariates

We considered a range of covariates to adjust the effects of exposure variable on outcome variables. The variables were selected in two stages. At first stage, we conducted a comprehensive literature search in several databases. Relevant studies conducted in LMICs, particularly in Asian countries, were summarized [2, 5, 6, 9, 13, 20, 23, 29–32]. Factors that were used to control the association between maternal anemia and adverse outcomes were listed. The availability of the listed variables was then checked with the survey we analysed and available variables were sorted out. Multicollinearity of the available variables was checked in the next stage and highly correlated variables were deleted. Finally, the selected variables were classified as individual-, household-, and community-level variables and adjusted in the models. The individual-level factors were maternal age (15–19, 20–24,25–29,30–34, 35–39, ≥40 years), maternal education and maternal working status (yes, no). Husband’s education (no education, primary, secondary and higher education), and household socio-economic status (poorest, poorer, average, richer, richest) were included as the household level factors. Community-level factors considered were the region of residence (Barisal, Chittagong, Dhaka, Khulna, Rajshahi, Rangpur, Sylhet) and place of residence (urban, rural).

### Statistical analysis

Descriptive statistics with mean (SE) and percentage were used to describe the characteristics of the respondents. The Chi-square test was used to explore the significance of the differences in anemia status across individual-, household-, and community-level factors. Mixed effect multilevel binary logistic regression analysis was used to determine the effects of exposure variable on outcome variables. Of the considered outcome variables, where the positive response was few (stillbirth, early neonatal mortality, perinatal mortality, preterm birth, prolonged labor), we used the multilevel Poisson regression model to determine the effects of exposure variable. Both unadjusted and adjusted associations were determined, separately for each of the outcome variables. In the unadjusted model, only a particular outcome variable was considered with the exposure variable. The association of exposure variable with outcome variables were adjusted for individual-, household-, and community-level factors in the adjusted models. The reason for using the multilevel model was the nesting structure of the BDHS data, where individuals were nested within a cluster and clusters were nested within a community. Previous studies found for this type of data mixed effect multilevel model produced better results [33, 34]. Results are reported as Odds ratio/Risk Ratio with its 95% Confidence Interval (95% CI). All analyses were two-sided and statistically significant was considered as *p<0*.*05*. All analyses accounted for the probability sample design. Stata version 15.10/SE (Stata Corporation, College Station, Texus, USA) was used for all statistical analyses.

## Results

### Sample characteristics

The background characteristics of the respondents are presented in Table 1. The mean age of the participant was 25.71 years, and the mean body mass index was 20.84 kg/m^2^. Around 44% of women were anemic. Nearly two-third (66%) of the total maternal women reported they faced pregnancy complications.

**Table 1:** Study population characteristics (N = 2259).

Characteristics	Mean (SD) or Percent (CI)
**Mean (SD)**	
Maternal age at birth (years)	25.71 (0.13)
Maternal weight (in kg)	47.55 (0.18)
Maternal height (cm)	150.94 (1.13)
Maternal body mass index (kg/m^2^)	20.84 (1.98)
Hemoglobin (g/dl)	120.71 (0.29)
**Percentage (95% CI)**	
Anemic	43.9 (41.3–46.4)
Pregnancy complication	65.6 (62.6–68.5)

SE, Standard error; 95% CI, 95% confidence interval

### Distribution of anemia across individual-, household-, and community-level characteristics

In Table 2, we presented distribution of the respondents across individual-, household-, and community-level characteristics considered. The majority of the respondents were in their ages 25–29 years at the time of their most recent pregnancy (26.1%) and were secondary educated (43.2%) at the time of the survey. Around one-third of the total women’s husbands were primary educated and 42% of the total women’s husbands were engaged in physical work. Around 68% of the total women analysed were included from rural areas.

**Table 2:** Distribution of the respondents by some selected socio-economic characteristics and anemia, (N = 2259).

Characteristics	Total maternal women (%)	Anemic maternal women	*p* values
**Maternal women’s age at most recent pregnancy (In years)**			
15–19	336 (14.9)	43.8 (37.8–49.9)	0.29
20–24	770 (34.1)	43.0 (38.8–47.3)	
25–29	593 (26.1)	41.5 (36.9–46.2)	
30–34	337 (14.9)	51.0 (44.7–57.3)	
35–39	157 (6.9)	42.7 (33.8–52.2)	
≥40	66 (2.9)	42.2 (29.1–56.5)	
**Maternal women’s education**			
None	411 (18.2)	48.5 (42.9–54.2)	0.017
Primary^1^	706 (31.2)	46.6 (42.3–51.0)	
Secondary^2^	976 (43.2)	41.1 (37.9–44.6)	
Higher	166 (7.4)	35.6 (27.4–44.7)	
**Maternal women’s working status**			
No	2023 (89.6)	43.3 (40.7–45.9)	0.17
Yes	236 (10.5)	48.6 (41.4–55.8)	
**Husband’s education**			
None	596 (26.4)	45.9 (41.2–50.8)	0.113
Primary^1^	716 (31.7)	46.5 (42.1–51.0)	
Secondary^2^	651 (28.8)	40.7 (36.6–45.1)	
Higher	296 (13.1)	39.4 (33.4–45.8)	
**Husband’s occupation**			
Agriculture	615 (27.2)	48.3 (43.82–52.9)	0.040
Physical	943 (41.7)	41.0 (36.9–45.2)	
Services	131 (5.8)	47.6 (38.0–57.5)	
Business	516 (22.8)	41.4 (36.5–46.4)	
Others	54 (2.3)	55.5 (40.2–69.7)	
**Wealth Index** ^2^			
Poorest	486 (21.5)	49.4 (44.2–54.6)	<0.001
Poorer	435 (19.2)	49.8 (44.3–55.4)	
Middle	435 (19.2)	44.2 (38.9–49.6)	
Richer	441 (19.5)	40.5 (35.3–45.8)	
Richest	462 (20.4)	33.2 (28.2–38.6)	
**Region of residence**			
Barisal	256 (11.3)	47.1 (40.1–54.2)	0.022
Chittagong	414 (18.3)	39.1 (33.9–44.4)	
Dhaka	369 (16.3)	46.8 (41.1–52.5)	
Khulna	281 (12.4)	35.7 (29.1–42.9)	
Rajshahi	292 (12.9)	43.8 (37.5–50.3)	
Rangpur	313 (13.9)	50.5 (44.5–56.4)	
Sylhet	313 (13.9)	43.8 (36.9–50.3)	
**Place of residence**			
Urban	723 (32.0)	38.0 (33.3–43.1)	0.010
Rural	1536 (68.0)	45.6 (42.7–48.6)	
**Total**	2259(100)	43.9 (41.3–46.4)	

Note: The numbers inside the parentheses represent the percentages,

^1^Primary completed is defined as completing grade 5,

^2^ Secondary completed is defined as completing grade 10,

^2^Wealth index measure the socio economic status. Proportions may not add to 100 due to missing data.

**Fig 1:**
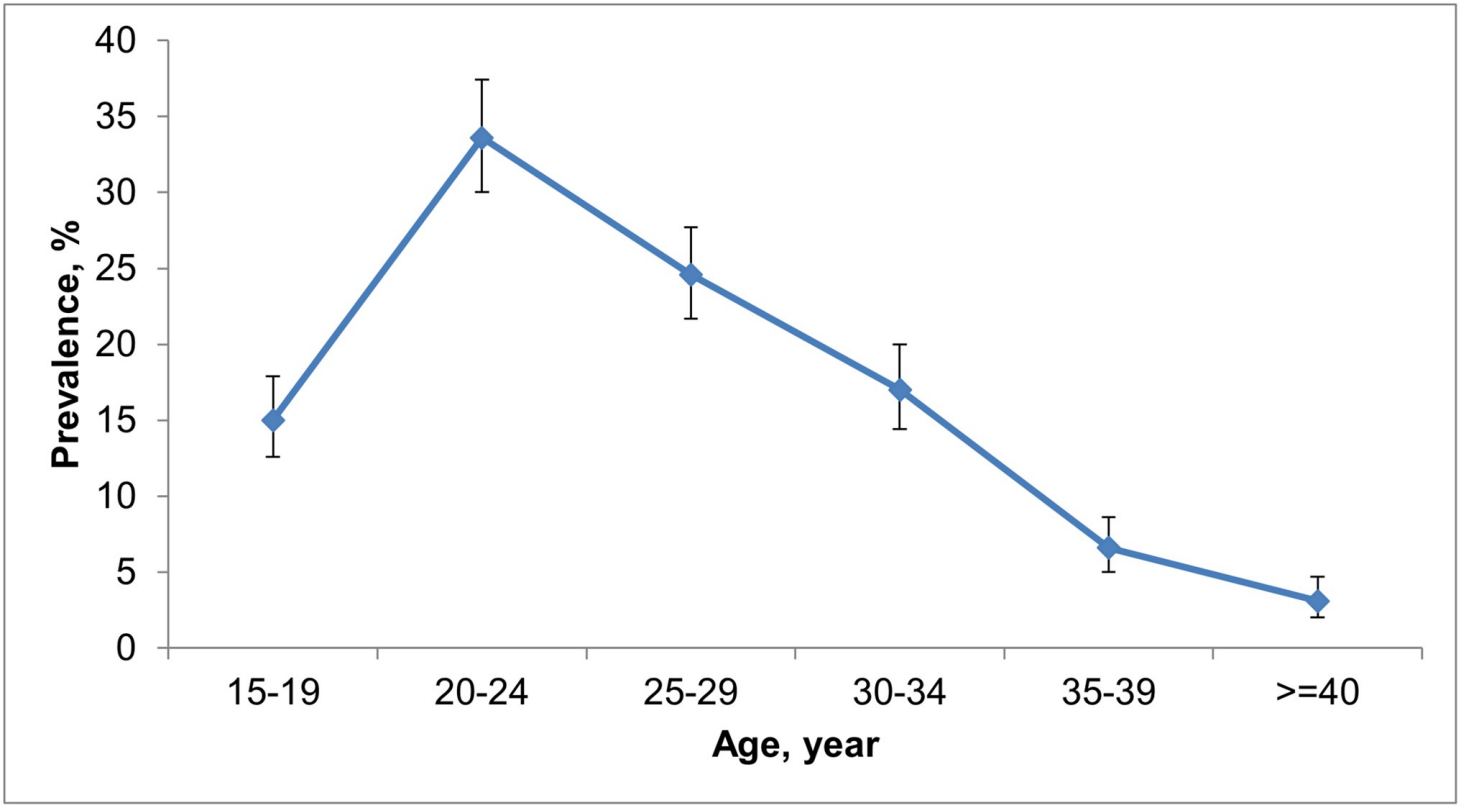
Prevalence of anemia by age group.

We also explored the difference in anemia status across individual-, household-, and community-level factors. The highest prevalence of anemia was observed among the women aged 20–24 years and then decreased as the age increased (Fig 1). Prevalence of anemia was found higher among women with comparatively lower education, comparatively lower husband’s education, and who resided in the households with comparatively lower wealth quintile. A higher prevalence of anemia was found among rural women as compared to urban women.

### Association between maternal anemia and adverse health outcomes

The associations between maternal anemia and adverse health outcomes are presented in Table 3. The likelihood of occurring pregnancy complications was found 1.39 times (AOR, 1.39, 95% CI, 1.09–2.41) higher among anemic women as compared to the non-anemic women. On the other hand, the likelihoods of becoming diabetic (AOR, 0.78, 95% CI, 0.49–0.98) and hypertensive (AOR, 0.79, 95% CI, 0.60–0.96) were found lower among anemic maternal women as compared to the non-anemic maternal women. Menstrual irregularities was found 21% lower (AOR, 0.79, 95% CI, 0.58–0.94) among anemic maternal women as compared to the non-anemic maternal women.

**Table 3:** Unadjusted and adjusted relation between maternal anemia and adverse maternal health outcomes, Bangladesh.

Health factors	Anemic group	N (n)	Proportion (%)	OR (95% CI)	
				Unadjusted	Adjusted
Pregnancy complication	Not anemic	874 (580)	66.4	1.00	1.00
	Anemic	614 (418)	68.1	1.07 (0.84–1.37)	**1.39 (1.09–2.41)**
Pregnancy termination	Not anemic	1241 (237)	19.1	1.00	1.00
	Anemic	913 (164)	17.9	0.96 (0.78–1.18)	0.95 (0.77–1.19)
Menstrual irregularities	Not anemic	1241 (307)	24.7	1.00	1.00
	Anemic	913 (290)	31.8	0.67 (0.56–0.79)	**0.79 (0.58–0.94)** **
Cesarean delivery	Not anemic	1241 (202)	16.3	1.00	1
	Anemic	913 (123)	13.5	0.85 (0.64–1.11)	1.16 (0.77–1.74)
Diabetes^1^	Not anemic	1218 (128)	10.5	1.00	1.00
	Anemic	912 (74)	8.1	0.75 (0.55–1.00)	**0.78 (0.49–0.98**)**
Hypertension^2^	Not anemic	1218 (325)	26.7	1.00	1.00
	Anemic	912 (203)	22.3	0.78 (0.60–0.99)	**0.79 (0.60–0.96)** **

Notes:

**p<0.05

The proportion (percent) and the result of three level logistic regression analysis (odds ratio (95% confidence interval)), are tabulated for each variable according to the anemia status. All models were adjusted by individual-, household-, and community level factors considered in this study.

### Association between maternal anemia and adverse birth outcomes

The associations between maternal anemia and adverse birth outcomes are presented in Table 4. We found 1.87 times (AOR, 1.87, 95% CI, 1.06–5.10) higher likelihood of early neonatal deaths among newborns of anemic mothers as compared to the newborn of non-anemic mothers. Similarly, the likelihood of preterm birth was found 2.03 times (AOR, 2.03, 95% CI, 1.01–4.25) higher among anemic mothers as compared to the non-anemic mothers. Around 1.54 (AOR, 1.54, 95% CI, 1.09–3.52) times higher likelihood of prolonged labor was also reported among anemic mothers as compared to the non-anemic mothers.

**Table 4:** Unadjusted and adjusted relation between maternal anemia and adverse birth outcomes, Bangladesh.

Birth outcomes	Anemic group	N (n)	Proportion (%)	OR /RR (95% CI)	
				Unadjusted	Adjusted
Low birth weight	Not anemic	1286 (233)	18.1	1.00	1.00
	Anemic	973 (178)	18.3	0.97 (0.76–1.23)	0.97 (0.77–1.22)
Stillbirths *	Not anemic	1286 (22)	1.7	1.00	1.00
	Anemic	973 (8)	0.8	0.99 (0.98–1.00)	0.45 (0.18–1.16)
Early neonatal death^*^	Not anemic	640 (7)	1.1	1.00	1.00
	Anemic	486 (11)	2.3	1.01 (1.0–1.03)	**1.87 (1.06–5.10)** **
Perinatal death*	Not anemic	1286 (29)	2.6	1.00	1.00
	Anemic	973 (19)	2.0	0.99 (0.98–1.00)	0.95 (0.49–1.86)
Preterm birth^*^	Not anemic	44 (13)	29.6	1.00	1.00
	Anemic	42 (17)	40.5	1.12 (0.91–1.37)	**2.03 (1.01–4.25)** **
Prolonged labor^*^	Not anemic	44 (13)	29.6	1.00	1.00
	Anemic	41 (11)	26.8	0.97 (0.80–1.18)	**1.54 (1.09–3.52)** **

Notes:

**p<0.05

The proportion (percent) and the result of three level logistic regression analysis (*Multilevel poisson regression). All models were adjusted by individual-, household-, and community level factors considered in this study.

## Discussion

The aim of this study was to determine the effects of maternal anemia on adverse maternal health and birth outcomes. For this, we analysed nationally representative cross-sectional 2011 BDHS data. Advanced statistical modelling was used to determine the targeted associations adjusted for the individual-, household-, and community-level factors. We found lower likelihoods of menstrual irregularities, diabetes and hypertension among anemic maternal women as compared to the non-anemic maternal women. Of the birth outcomes considered, higher likelihoods of early neonatal death, preterm birth, and prolong labor were found among newborns of the anemic maternal women as compared to the non-anemic maternal women. These findings are robust as they were generated by analyzing the nationally representative data with a range of confounding factors adjusted. Therefore, the findings will be helpful for the policy and program makers to make evidence-based policies and programs to reduce the prevalence of these adverse outcomes.

In this study, we found the prevalence of anemia among Bangladeshi women was 43.9%. Apart from the high prevalence of anemia at the national level, the degree of prevalence varies over some selected socio-demographic characteristics. Household economic status measured by the wealth index is an important factor related to the variation of anemia prevalence, with poor women being more likely to anemic than richer women. This finding is consistent with the findings from the other studies in LMICs [20, 35–37]. Prevalence of anemia varied substantially by maternal educational status and a higher prevalence was observed among primary and secondary educated maternal women. This may indicator of the improvement in education continuation rate up to the secondary level among the lower socio-economic classes in which anemia is still persists. Several studies in LMICs, including Pakistan [31], India [38], China [32], found a significantly higher prevalence of anemia among the women with no formal education.

This study identified around 68% anemic women had had pregnancy complications. The likelihood of pregnancy complications was found 1.39 times higher among anemic maternal women as compared to the non-anemic maternal women. We could not validate our findings because of the lack of relevant literature. The possible mechanism is anemia during pregnancy leads to the suboptimal oxygen and nutrients supply to the placenta [39]. This led to the abnormal placenta and the subsequent placental function being impaired. This then works as the key to adverse health complications. There is evidence that maternal health complications are responsible for around 30% of the total perinatal deaths [40], 20% of the total maternal mortality, and 3.4 million disability-adjusted life years [41] in LMICs. Our study finding regarding maternal anemia and pregnancy complications suggests that reduction of anemia prevalence could be an effective pathway to reduce these adverse birth outcomes. Screening for, and effectively treating complications in anemic women might therefore result in reduced adverse maternal and perinatal outcomes.

We found a higher likelihood of preterm birth among anemic maternal women as compared to the non-anemic maternal women. Every year an estimated 15 million babies are born preterm, around 3% of them are born in Bangladesh which is equivalent to 14% of the total live births in Bangladesh [42]. This ranked Bangladesh as the 7^th^ larger number of preterm birth countries in the world (N = 424,144) [42]. Such a higher prevalence of preterm birth is reported as a major cause of child mortality in LMICs, including Bangladesh, with around 1 million deaths in a year [43]. Previous studies found that women with low educational status, low number of antenatal visits, previous history of child death, low socioeconomic status and underweight as the risk factor for preterm birth [44, 45], whereas in the current study we reported maternal anemia is a significant risk factor for maternal anemia. Our finding was consistent with a recent meta-analysis [46], which conclude that first and second-trimester anemia was associated with the increased risk of preterm birth. Reported three major postulated biological mechanisms of preterm birth were maternal infection, hypoxia and oxidative stress [47]. Anemia increases the occurrence of maternal and fetal stress [48]. The underlaying pathway is to increase low grade chronic hypoxia because of iron deficiency [48]. This in turn can initiate labour and eventually result in preterm birth [47, 49]. There is also evidence that preterm birth increases the risks of perinatal mortality and early neonatal mortality. This linkage is also indicative of the higher risk of early neonatal mortality and perinatal mortality among newborns of the anemic maternal women as compared to the non-anemic maternal women. This observation is consistent with our study findings as well as other studies in LMICs [29, 50–52].

This study found lower risk of menstrual irregularities among anemic maternal women. We are not able to justify our findings due to the lack of evidence in the literature. However, there is evidence that anemia is a significant contributor of insulin resistance [53–55]. Insulin resistance is associated with type two diabetes mellitus, hypertension and dyslipidemia [56] which are usually more common among overweight and obese women [57], among which anemia is less prevalent [58]. However, in this study, lower prevalence of diabetes and hypertension were found among anemic as compared to non-anemic women. Previous studies reported contradicting findings regarding these associations [59–61]. The reason behind such contradictory results may be the variations in socio-economic conditions across several study settings. Early marriage, repeated pregnancy, poor dietary habits, poverty and illiteracy are all well-established factors of prevalence and severity of anemia [62]. Many other previous studies identified these factors as the opposite of developing diabetes and hypertension [63, 64].

### Strength and limitations

This study has several strengths and a few limitations that should be acknowledged. To the best of our knowledge, this is the first nationally representative study that determines the association of maternal anemia with a range of adverse maternal health and birth outcomes. This study includes appropriate statistical models with a range of individual-, household-, and community-level covariates to establish the associations. Consequently, the findings are more appropriate and should be used for national-level policies and programs making. The major limitation of this study is the analysis of cross-sectional data. Therefore, the findings are correlational only, not casual. Moreover, other than the covariates adjusted in the models, environment and health facility level factors play an important role on adverse health and birth outcomes. However, we could not adjust these variables in this study because of lack of relevant data in the survey we analysed in this study. Additionally, anemia status should be measured prospectively, i.e., before or during pregnancy, to examine its adverse effects on the subsequent months. However, anemia status reported in the BDHS was recorded retrospectively. This may affect the findings reported in this study. However, any distortion is likely to be random. The use of longitudinal data should be considered to address this limitation. However, we are not aware of any longitudinal data sources in Bangladesh that should be used to address these limitations.

## Conclusion

Anemia during pregnancy is common in Bangladesh with around 44% prevalence. Maternal anemia was found to be associated with an increased likelihood of pregnancy complications and decreased likelihoods of menstrual irregularities, diabetes, and hypertension. The likelihoods of preterm birth, perinatal mortality and early neonatal mortality were also found higher among newborns of anemic maternal women as compared to the newborns of non-anemic maternal women. This indicates maternal anemia is one of the major burdens to improving maternal and child health as well as reductions of related adverse outcomes. As such, maternal anemia should be considered an important challenge to achieving the health-related SDGs by 2030. Awareness-building programs regarding the adverse effects of maternal anemia on health and birth outcomes at the community level should be considered. The government should also take the initiative to provide iron tablets during pregnancy for all women.
